# Impact of Digital Economy Development on Carbon Emission Efficiency: A Spatial Econometric Analysis Based on Chinese Provinces and Cities

**DOI:** 10.3390/ijerph192214838

**Published:** 2022-11-11

**Authors:** Liang Liu, Yuhan Zhang, Xiujuan Gong, Mengyue Li, Xue Li, Donglin Ren, Pan Jiang

**Affiliations:** 1 School of Economics and Management, Southwest University of Science and Technology, Mianyang 621010, China; 2 School of Environment and Resource, Southwest University of Science and Technology, Mianyang 621010, China

**Keywords:** digital economy, carbon emission efficiency, spatial effects, super-SBM-undesirable model

## Abstract

In the realistic context of the development of China’s digital economy and carbon peaking and carbon neutrality goals, to efficiently achieve high-quality economic and green and low-carbon transformation, this paper investigates the impact of digital economy development on the carbon emission efficiency of 30 Chinese provinces and cities from 2011–2019. In this paper, firstly, the digital economy development index and carbon emission efficiency are calculated by the entropy method and the Super-SBM-Undesirable Model. Secondly, the Spatial Lag Model (SAR) and the Spatial Durbin Model (SDM) are respectively constructed under the adjacency matrix and the geographic distance matrix to empirically test the spatial impact of the digital economy on carbon emission efficiency. The results show that: the digital economy development and carbon emission efficiency of Chinese provinces and cities both show the spatial distribution characteristics of stronger in the East and weaker in the Middle and West; the digital economy development in Chinese provinces and cities has a significantly positive direct and spatial spillover effect on carbon emission efficiency; there are differences in the direct and spatial spillover effects of various dimensions of the digital economy development on the carbon emission efficiency in Chinese provinces and cities; the direct effect of the digital economy development on the carbon emission efficiency in Chinese provinces and cities has significant regional heterogeneity among eastern, central, and western regions. This paper provides new empirical evidence for developing countries such as China to proactively develop a digital economy to promote energy conservation and emission reduction to realize green and low-carbon transformation.

## 1. Introduction

Energy conservation, emission reduction, and green development are the essential backbones for promoting carbon peaking and carbon neutrality goals. In recent years, the vigorous development of digital technologies such as the Internet and artificial intelligence has deepened the integration of the digital economy with the real economy, generated green production and consumption patterns, strengthened the environmental protection concept of low-carbon life, and released significant green benefits in terms of social and economic development. The decarbonization of the economy has become a global development trend [1]. Focusing on China, at the end of 2021, China’s State Council issued a plan to facilitate the development of the digital economy in the 14th Five-Year Plan period (2021–2025), which proposed to continuously promote the construction of green digital centers, accelerate the application of intelligent energy construction, and accelerate the low-carbon transformation of the energy industry [2]. During the same period, the China Academy of Information and Communications Technology(CAICT) released the White Book of Digital Carbon Neutrality, pointing out that the deep integration of digital technology and focused carbon emission areas can reduce energy and resource consumption and promote energy and cost optimization of traditional industries [3]. The in-depth application of digital technology in the development of the digital economy provides an important impetus for China’s low-carbon transition and the achievement of the “double carbon” goal.

Prevailing research has studied carbon emission reduction from the perspective of digital technology and the digital economy development. On the one hand, the deep integration of digital technology with socio-economic activities can directly reduce energy consumption [4] or indirectly achieve carbon emission reduction by weakening the increasing effect of energy consumption structure on regional carbon emissions [5]. For example, the popularity and utilization of the Internet brought about by the development of information and communication technologies can promote carbon emission reduction [6], and the development of Internet technology would reduce environmental pollution by improving production efficiency and energy efficiency [7]. In addition, smart city construction can achieve regional energy conservation and emission reduction by improving energy efficiency [8]. On the other hand, the development of a digital economy contributes to influencing carbon productivity [9] as well as energy consumption structure and utilization efficiency by optimizing the resource allocation structure, improving the utilization rate of production resources [10], upgrading industrial structure [11], and promoting green technology innovation, which in turn reduces the carbon emission intensity [12]. However, some studies have argued that the digitization of the economy will impose a heavier burden on the environment [13], and the speedy development of digital industries will lead to a rapid increase in energy consumption, and consequently results in increased carbon emissions [14]. Although there have been abundant studies on the relationship between the digital economy and carbon emissions which have confirmed the spatial heterogeneity between them [15], no study has yet deeply analyzed the relationship between the impact of the digital economy on carbon emission efficiency. As an essential evaluation criterion for low-carbon economic development, carbon emission efficiency reflects the coordination between economic growth and sustainable development [16]. Therefore, it would be more sensible to incorporate carbon emission efficiency into the research framework to reflect the function of digital economy to promote green and low-carbon sustainable development.

Based on 30 provinces and cities in China, this paper uses a spatial econometric model to explore the relationship between digital economy development and regional carbon emission efficiency. By applying the entropy method and the Super-SBM-Undesirable Model to separately calculate the digital economy development index and the carbon emission efficiency of provinces and cities from 2011–2019, this paper constructs a spatial weight matrix and a spatial econometric model to conduct an empirical test of the influence and the spatial spillover effect that digital economy development has on carbon emission efficiency. Compared with existing studies, the novelties of this paper are firstly, digital economy development and carbon emission efficiency are included in the same research framework; secondly, the distribution characteristics of digital economy development and carbon emission efficiency of Chinese provinces and cities are visualized and analyzed; finally, the spatial spillover effects of different digital economy development dimensions on carbon emission efficiency are considered.

## 2. Theoretical Analysis

### 2.1. The Digital Economy Development and Carbon Emission Efficiency

As an essential part of economic development, the digital economy not only provides new opportunities for economic growth but also plays a vital role in carbon peaking and carbon neutrality goals. With the development of the economy, more and more cities have achieved the absolute decoupling of economic growth with carbon emissions and achieved the transition to green and low-carbon development [17]. A digital economy can promote carbon emission reduction by enhancing innovation capacity, alleviating financing constraints [18], promoting industrial structure upgrading [19], and increasing investment in R&D [20], etc. Transportation is an influential area for controlling carbon emissions, and the choice of travel means, and the increase in road vehicles have a direct impact on the total regional carbon emissions [21,22]. However, the combination of the digital economy and transportation industry has not only driven the development of sharing economy and reduced carbon emissions in the transportation field but also created new momentum for socio-economic development. Especially in the distribution business of the logistics industry, the use of shared new energy vehicles has reduced the total distribution distance, improving distribution efficiency while reducing energy consumption and carbon emissions [23]. Moreover, digital technology not only contributes to environmental protection but also to human health. In the face of increasingly serious air pollution and the public health hazards it generates, the development and application of new air quality monitoring equipment and drone monitoring technology have provided accurate and efficient decision support for the government to develop environmental protection measures and promote the sustainability of society [24,25].

Existing research suggests that the digital economy can also positively impact high-quality new energy development. In order to be specific, with every 1% increase in the digital economy index, high-quality new energy development will increase by an average of 0.191% [26]. The digital economy can stimulate the transition to renewable energy by improving the governance capacity of governments [27]. Meanwhile, an IT-focused digital economy not only helps to provide solutions for developing clean energy in low-carbon societies [28] but also helps to optimize energy structure, improve energy use efficiency [5,29], and reduce energy consumption. For example, the development of the sharing economy, such as Uber and Car-sharing [30], can effectively reduce energy costs and waste [31], further promote the energy revolution, and improve energy efficiency. In addition, digital technology also contributes to the development of the labor market. On the one hand, digital technology provides a more professional human resource pool for the labor market, and the depth and breadth of university students’ knowledge of cutting-edge digital technologies such as Artificial Intelligence, Internet of Things, and Edge Computing will have an influential impact on the development of the industry, as well [32,33]. On the other hand, digital technology can provide a more flexible and convenient means of working and communicating for employees and more extensive access to recruitment information for job seekers. By such a means, the efficiency of the labor market and the match between laborers and jobs can be prominently improved [34].

To summarize, although there is little study on the relationship between the digital economy and carbon emission efficiency, from the perspective of inputs and outputs, the digital economy helps to improve energy utilization efficiency as well as social labor production efficiency [35], which helps improve socio-economic development and promote carbon emission reduction. Therefore, we tentatively conclude that the development of the digital economy contributes to carbon emission efficiency.

### 2.2. Spatial Spillover Effects of the Digital Economy Development

Tobler’s First Law of Geography assumes that things in proximity are more closely related [36], and both the development of the digital economy and digital technology innovation in China show significant spatially dependent characteristics [37,38]. The digital economy has broadened the information interaction channels and reduced spatial and temporal limitations of resource elements, which enhanced the breadth and depth of socio-economic activities between regions [39]. The digital economy realizes the efficient allocation of innovation factors and breaks the spatial and temporal barriers to mutual collaboration among innovation subjects, and improves regional innovation capacity and innovation efficiency through knowledge spillover [40].

As for promoting green development, the digital economy has positively influenced haze pollution management, and the inhibitory effect of digital economy development on haze pollution is even more significant in eastern China [41]. In addition,, the digital economy can promote regional clean energy development [42] and enhance green total factor productivity [43], with positive spatial spillover effects on neighboring regions. As for achieving carbon emission reduction, the digital economy helps to improve regional carbon productivity and release positive external influences on neighboring areas through the well-developed local low-carbon transformation [44]. The development and application of digital technologies can reduce the carbon emission intensity of neighboring regions via spatial spillover effects and promote the formation of regional coordinated development of carbon emission reduction patterns [45]. Meanwhile, the promotion effect of the digital economy on carbon emission reduction in Chinese provinces and cities shows regional heterogeneity, with the strongest suppressive effect in the central region and the western and eastern regions following [19]. Visibly, the impact of the digital economy on carbon emission reduction has significant differences in resource endowments, city sizes, and innovation capabilities [46]. Thus, we tentatively conclude that there is a spatial spillover effect of the impact of the digital economy on regional carbon emissions.

## 3. Variable Selection and Data Description

### 3.1. Variable Definition

#### 3.1.1. Explained Variable Carbon Emission Efficiency (CEE)

This paper uses Matlab software to measure the carbon emission efficiency of 30 Chinese provinces and cities based on the Super-SBM-Undesirable Model. Table 1 shows the selected input-output indicators. This paper chooses input indicators from labor, capital, and energy levels, the desired output indicators from the economic development level, and the non-desired output indicators from the carbon emission level. Specifically, the labor input indicator is the number of employed persons, and the capital input indicator is the capital stock calculated using the perpetual inventory method (depreciation rate of 10.96%) [47], with 2011 as the base period and the energy input indicator is the total energy consumption. The desired economic output indicator is the real GDP calculated using 2011 as the base period, while the undesired carbon output indicator is the total carbon dioxide emissions from all energy sources.

#### 3.1.2. Explanatory Variable the Digital Economy Development Level (Digital)

This paper calculates the digital economy development index of provinces or cities from 2011–2019 by establishing the digital economy development index system and using the entropy method [19]. On the basis of referring to existing research [19,48,49], this paper constructs a digital economy development index system including 15 positive indicators from four dimensions: digital infrastructure, Internet development, digital industry development, and digital finance. By using the entropy weight method, this paper calculates the weights of each indicator (as shown in Table 2) as well as the digital economy development index of the study sample.

#### 3.1.3. Control Variables

Based on the STIRPAT Model, this paper first identifies control variables at three levels: population (Population), property (Affluence), and technology (Technology). Of these variables, the population-level variable measured by the number of people per unit area is population density (PopuD), the property-level variable measured by GDP per capita is economic development (PGDP), and the technology-level variable measured by total internal expenditure on R&D is technological innovation (RD). Moreover, according to existing studies, differences in regional industrial structure can directly or indirectly affect carbon emission intensity and efficiency [50,51]. In addition, trade openness can affect carbon emissions positively or negatively [19], while the changes in consumption patterns caused by urbanization rates will lead to an increase in carbon emissions as well [52]. Therefore, this paper incorporates the above variables and defines the industrial structure (Indus) as the ratio of tertiary industry output to secondary industry [53], openness to foreign trade (Open) as the ratio of total foreign investment imports and exports to regional GDP [19], and urbanization (Urban) as the ratio of non-agricultural population to the total population [54].

This paper selects panel data of 30 provinces and cities in China from 2011–2019 for empirical study (Tibet, Hong Kong, Macau, and Taiwan are not included in the study sample due to data availability). The data in this paper are obtained from the China Statistical Yearbook, China Energy Statistical Yearbook, China City Statistical Yearbook, the statistical yearbooks of Chinese provinces and cities, and the CSMAR database. The research variables and their definitions are shown in Table 3.

### 3.2. Model Construction

#### 3.2.1. Super-SBM-Undesirable Model

To overcome the shortcomings of the traditional Data Envelopment Analysis (DEA Model) based on the radial distance function for efficiency measurement, Tone [55] proposed a non-radial and non-angle SBM Model (Slacks-Based Measure), which effectively solves the slackness problem. However, the efficiency measured by the SBM Model takes the value of (0,1], and there may be multiple decision units with the efficiency value of 1, which leads to the inability to compare and evaluate the fully efficient decision units. On this basis, Tone further proposes the super SBM model (super SBM), which further decomposes the decision units with an efficiency value of 1 so that the efficiency value is not limited to the interval of (0,1], circumventing the problem of complete efficiency of multiple decision units and realizing the mutual comparison among effective decision units. Therefore, according to the research objectives, this paper selects the Super-SBM-Undesirable Model to measure the carbon emission efficiency values of 30 Chinese provinces and cities, which are calculated as follows.

(1) $$\rho =\min\frac{1+\frac{1}{m}\sum_{i=1}^{m}\frac{s_{i}^{x}}{x_{i0}}}{1-\frac{1}{s_{1}+s_{2}}\left(\sum_{k=1}^{s_{1}}\frac{s_{k}^{y}}{y_{k0}}+\sum_{l=1}^{s_{2}}\frac{s_{l}^{z}}{z_{l0}}\right)}\;s.t.\;x_{i0}\geq \sum_{j=1,\neq 0}^{n}\lambda_{j}x_{j}-s_{i}^{x},\forall i;\;y_{k0}\leq \sum_{j=1,\neq 0}^{n}\lambda_{j}y_{j}+s_{k}^{y},\forall k;\;z_{l0}\geq \sum_{j=1,\neq 0}^{n}\lambda_{j}z_{j}-s_{l}^{z},\forall l;\;1-\frac{1}{s_{1}+s_{2}}\left(\sum_{k=1}^{s_{1}}\frac{s_{k}^{y}}{y_{k0}}+\sum_{l=1}^{s_{2}}\frac{s_{l}^{z}}{z_{l0}}\right)>0;\;s_{i}^{x}\geq 0,s_{k}^{y}\geq 0,s_{l}^{z}\geq 0,\lambda_{i}\geq 0,\forall i,j,k,l.$$

In the above equation, *n* denotes the number of decision units, and *m* denotes the number of input indicators. Variate *s*_1_ denotes the number of desired output indicators, *s*_2_ denotes the number of non-desired output indicators, $s_{i}^{x}$, $s_{k}^{y}$ and $s_{l}^{z}$ are the corresponding slack variable, and $\lambda_{i}$ is the weight vector.

#### 3.2.2. Econometric Model Construction

1. Baseline model

In this paper, we first construct the following OLS model to analyze the impact of digital economy development on regional carbon emission efficiency without considering the influence of spatial factors.

(2) $$CEE_{it}=\alpha_{0}+\alpha_{1}Digital_{it}+\alpha_{2}Controls_{it}+Space_{i}+\varepsilon_{it}$$

In this equation, *i* denotes Chinese provinces and cities, and *t* denotes the year, *CEE*, *Digital*, and *Controls* respectively denote the explained variable carbon emission efficiency, the explanatory variable digital economic development, and the six control variables, Variate *α*_0_ is a constant term, *α*_1_ is the estimated parameter of the explanatory variable, and *α*_2_ is the estimated parameter of the control variable. Variate *Space_i_* denotes the spatial fixed effect, and *ε_it_* is a random error term.

2. Spatial econometric model

Traditional econometric models consider variables to be independent of each other in spatial dimensions and fail to consider the spatial dependence of variables [56]. However, there is a high spatial dependence between digital economic development and carbon emission efficiency. So, taking spatial factors into consideration, this paper constructs a spatial econometric model to carry out further analysis. Currently, spatial models include the Spatial Lag Model (SAR), the Spatial Error Model (SEM), and the Spatial Durbin Model (SDM). Generally, the SAR model is used when the explained variable has spatial dependence. As for the SDM model, it is used when the residual term has spatial dependence, and the SDM model is used when both explained and explanatory variables have spatial dependence. In this paper, we mainly adopt SAR and SDM models for empirical study and construct the SAR and SDM models of model (2) as follows.

(3) $$CEE_{it}=\rho WCEE_{it}+\beta_{1}Digital_{it}+\beta_{2}Controls_{it}+Space_{i}+\varepsilon_{it}$$

(4) $$CEE_{it}=\rho WCEE_{it}+\gamma_{1}Digital_{it}+\gamma_{2}Controls_{it}+\gamma_{3}WDigital_{it}+\gamma_{4}WControls_{it}+Space_{i}+\varepsilon_{it}$$

Variate *ρ* is the spatial regression coefficient, and *W* is the *n* × *n* spatial weight matrix. Variate *β* and *γ* are the estimated parameters of the variables, and the remaining variables have the same meaning as above.

3. Spatial weight matrix

Due to the vast size of China, this paper focuses on the influence of geographic location factors and chooses the adjacency matrix and the geographic distance matrix. Following the traditional definition, provinces and municipalities that are geographically adjacent, i.e., share a common border, are given a value of 1 in the adjacency matrix and 0 otherwise. In addition, the geographic distance matrix is constructed as the inverse of the straight-line geographic distance between provincial capitals or municipalities. In addition, to test the robustness of the effect of regional digital economy development on carbon emission efficiency, the inverse distance square matrix and the economic-geographic distance nested matrix are selected for subsequent robustness analysis, with consideration of both economic and geographic factors.

### 3.3. Spatial Weight Matrix

The descriptive statistics of the variables are shown in Table 4. As can be seen from the table, the mean value of CEE is 0.4505, and the standard deviation is 0.2218, which indicates that there is a significant difference in the carbon emission efficiency among 30 Chinese provinces and cities. Meanwhile, the mean value of Digital is 0.1156, and the standard deviation is 0.0991, suggesting that there are also some differences in the development of the digital economy among 30 Chinese provinces and cities. Therefore, it is feasible to consider individual differences among the study samples by using a spatial econometric model, that is, to consider the influence of spatial factors on the development of the digital economy and carbon emission efficiency.

**Figure 1:**
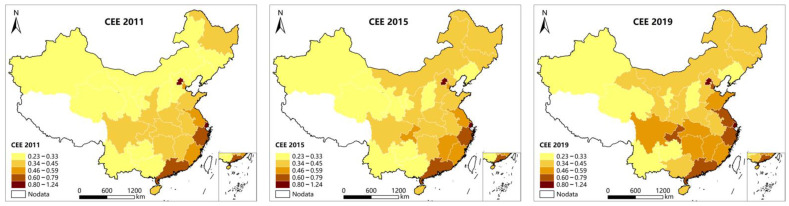
Spatial layout of the carbon emission efficiency in 30 Chinese provinces.

**Figure 2:**
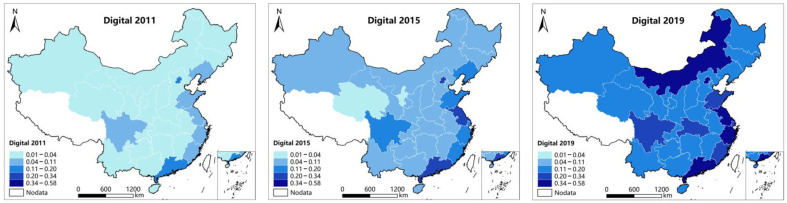
Spatial layout of the digital economy index in 30 Chinese provinces.

In order to visualize the Spatial pattern evolution of Chinese carbon emission efficiency and the digital economy development, this paper uses ArcGIS to map the spatial layout of carbon emission efficiency and digital economy indices of the study sample in 2011, 2015, and 2019. As is shown in Figure 1, the carbon emission efficiency of China has improved substantially in general during the study period, and the carbon emission efficiency of the eastern region is much higher than that of the central and western areas. Although the carbon emission efficiency of Jiangxi, Hunan, and Hubei in central China, as well as Sichuan and Chongqing in western China, is relatively higher than other provinces and cities in the area, it is still lower than that of the eastern coastal region. Up to 2019, the carbon emission efficiency of China shows an obvious spatial correlation. As can be seen in Figure 2, the digital economy of Chinese provinces and cities has been flourishing during the study period of the sample, presenting the characteristic of the stronger in the East and weaker in the Middle and West. As a new highland of development in the west, Sichuan Province has a developed communication infrastructure and rich industrial digital transformation needs, so its digital economy development results are particularly outstanding among the western regions. Up to 2019, there is a spatial correlation between the digital economy development of Chinese provinces and cities, especially in eastern China. The uneven and spatially correlated pattern distribution characteristics of the carbon emission efficiency and the digital economy development of China ensure the variability among research samples and the applicability of research method selection.

## 4. Spatial Econometric Regression

### 4.1. Spatial Dependence Test

Before applying the spatial econometric model, it is necessary to test the spatial dependence of the variables. This paper uses Moran’s I to test spatial dependence and correlation of carbon emission efficiency. Moran’s I value ranges from −1 to 1, and the higher the absolute value is, the stronger the correlation is shown. If the value is greater than 0, it indicates that the variable has positive spatial autocorrelation, and if the value is less than 0, it indicates that the variable has negative spatial autocorrelation, and if the value is equal to 0, it indicates that the variable has random distribution in space.

The global Moran’s I of CEE from 2011 to 1019 under the four spatial weight matrixes is shown in Table 5. The results show that Moran’s I am significantly positive at the 5% confidence level, regardless of the matrix, indicating that China’s CEE shows significant positive spatial dependence, which means that the carbon emission efficiency of provinces and cities is influenced by the associated. Thus, spatial effects should be considered when studying carbon emission efficiency.

**Figure 3:**
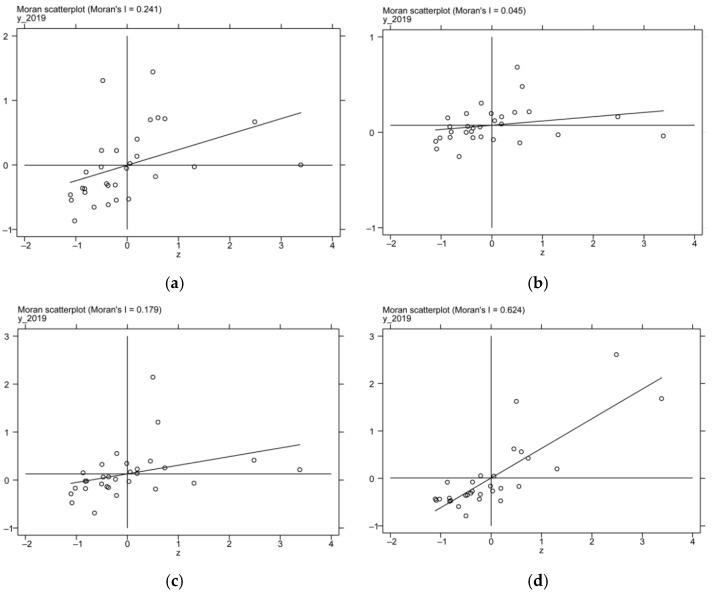
(**a**) Local Moran diagram of 2019 CEE under the adjacency matrix; (**b**) Local Moran diagram of 2019 CEE under the geographic distance matrix; (**c**) Local Moran diagram of 2019 CEE under the inverse distance square matrix; (**d**) Local Moran diagram of 2019 CEE under the economic-geographic distance nested matrix.

To further verify the spatial dependence of CEE, this paper plots the local Moran test results of this variable under four matrixes in 2019 using Stata15.1 software (as shown in Figure 3). It can be found that the high-high and low-low clustering characteristics of the carbon emission efficiency in China are apparent. It not only reflects the unbalanced development of carbon emission efficiency in Chinese provinces and cities but also intuitively shows the positive spatial correlation of CEE, further confirming the applicability of the spatial econometric model in this study.

### 4.2. Analysis of Regression Results

#### 4.2.1. Baseline Model Selection

This paper first exhibits the baseline regression results of the non-spatial panel data of model (1) and determines whether it should be a fixed-effects model by the Hausman test. Since this paper focuses on the effect of geographical factors and the sample period is smaller than the number of cross sections, the spatial fixed effects model is selected [57]. The Hausman test results reported in Table 6 reject the null hypothesis, so this paper uses the fixed effects model for the subsequent analysis.

In order to determine the appropriate spatial econometric model, this paper first performs the LM and robust LM test on the non-spatial panel model [58]. The test criteria are as follows: if both LM statistics are insignificant and the spatial econometric model is inappropriate for the study, then the OLS model will be chosen. If the LM-lag statistic is significant, the SAR model will be selected. In addition, if the LM-error statistic is significant, the SEM model will be applied. If both statistics are significant, compare the robust LM statistics as above. Then if robust LM statistics are both significant, construct the SDM model and perform the Wald test and LR test. In addition, if the LM statistics are inconsistent with the model pointed out by the Wald statistics or LR Statistics, the SDM model will be chosen as the general model of the SAR and SEM models [59].

The results of the spatial econometric model applicability tests for the four matrixes are shown in Table 7. The results show that under the adjacency matrix, LM-error, Robust-error, and LM-lag are all significant at the 1% confidence level, but Robust LM-lag is not significant, indicating it is appropriate to select the SEM model. However, the Wlad statistic accepts the null hypothesis of “SDM model can degenerate to SAR”. Thus, in the case of inconsistent points of statistics, it is suitable to use the SDM model. Meanwhile, under the geographic distance matrix and the inverse distance square matrix, the LM test and robust LM test results are both significant, and the Wald statistic accepts the null hypothesis of “SDM model can degenerate to SAR model”. So, it is suitable to choose the SAR model under these two matrixes. In addition, under the economic-geographic distance nested matrix, both the LM test and robust LM test results are significant, and neither the Wald test nor the LR test rejects the null hypothesis that the SDM model degenerates to SAR or SEM model. So, it is suitable to use the SDM model under this matrix. In summary, this paper selects the spatial fixed effect SDM model under the adjacency matrix (W_binary_) and the economic-geographic distance nested matrix (W_dis&eco_) and selects the spatial fixed effect SAR model under the geographic distance matrix (W_distance_) and the inverse distance square matrix (W_distance2_) for subsequent analysis.

#### 4.2.2. Spatial Econometric Regression Results

The spatial econometric regression results of the SDM and SAR models are shown in Table 8. The spatial coefficients ρ of both the adjacency matrix and the geographic distance matrix are significantly positive at the 1% confidence level with coefficients of 0.195 and 0.454, respectively, indicating the significant positive spatial spillover effect on the carbon emission efficiency of Chinese provinces. Between provinces and cities with geographical borders or similar distances, resource factors such as talent, material, and financial flow more conveniently and efficiently, and the development level of science and technology, industrial structure, and energy consumption structure between them are more similar, so the provinces and cities with high carbon emission efficiency can drive the co-improvement of carbon emission efficiency in the surrounding regions.

The explanatory variable digital economy development (Digital) is significantly positive at the 1% confidence level, indicating that the regional digital economy development has a significant positive contribution to improving carbon emission efficiency. As a new economic development model, the digital economy can efficiently allocate workforce and improve technical efficiency through digital technologies applications, such as artificial intelligence, to empower high-quality economic development [60]. While on the other hand, it can upgrade the industrial structure by promoting the digitalization and intelligent transformation of industries [61], which contributes to the efficiency of energy use and reduces carbon emissions, and in turn realizes the improvement of carbon emission efficiency.

The regression results of the control variables show that both population density (PopuD) and openness to the outside world (Open) are significantly positive, indicating that the increase in population density of provinces and cities contributes to the efficiency of carbon emissions. On the one hand, provinces and cities with higher population density may have more developed and convenient public transportation networks, which reduce people’s commuting time to work and improve labor productivity and socio-economic benefits. On the other hand, provinces and cities with higher population densities have more developed and aggregated productive service industries, which helps to promote industrial structure optimization, improve energy use structure, and reduce carbon emission, thus improving carbon emission efficiency [62]. At the same time, the increase in openness helps to improve carbon emission efficiency, indicating that the impact of foreign direct investment on carbon emissions of Chinese provinces and municipalities supports the Pollution Halo Hypothesis [63]. The openness to the outside world not only helps to broaden access to resources but also helps governments to learn and absorb successful environmental management experiences and improve their environmental management systems while enhancing regional production levels, thus promoting the improvement of carbon emission efficiency. Urbanization (Urban) is significantly positive under the geographic distance matrix, indicating that urbanization also contributes to improving carbon emission efficiency, which may result from the fact that the increase in resident population in provinces and cities with higher urbanization rates or in the late stage of urbanization not only improves productivity but also promotes the upgrading of consumption structure. Meanwhile, technological progress and industrial agglomeration optimize industrial structure and improve energy utilization efficiency, which enhances local carbon emission reduction and emission efficiency.

#### 4.2.3. Decomposition of Spatial Spillover Effects

In order to analyze the spatial effects of the variables in detail, this paper further explores the decomposition results of the direct, indirect, and total effects estimated by the SDM and SAR models. The direct effect reflects the influence of the regional explanatory variables on the locally explained variables and the feedback influence on the locally explained variables, resulting from the local explanatory variables’ effect on the adjacent explained variables. The indirect effect reflects the effect of the neighboring explanatory variables on the local explanatory variables. The total effect is the sum of the direct and indirect effects.

The empirical results in Table 9 show that the direct effect of the digital economy development is significantly positive at the 1% confidence level, indicating that the digital economy development of provinces and cities contributes to local carbon emission efficiency improvement. It includes both the direct effect of the digital economy development on local carbon emission efficiency and the feedback mechanism that the digital economy development influences the carbon emission efficiency of neighboring areas and then impacts local carbon emission efficiency. The indirect effect is significantly positive at the 5% confidence level, indicating that digital economy development has a positive spatial spillover effect, which means the digital economy development also contributes to the neighboring regions’ carbon emission efficiency. It may be due to the effects of optimizing the resource allocation structure, improving energy utilization efficiency [11], and promoting the development of low-carbon industries generated by the digital economy development, which has a radiating effect on the development of the surrounding areas and thus boosts the overall carbon emission efficiency of the region.

Moreover, the decomposition results of the spatial spillover effects of the control variables show that under the adjacency matrix, economic development (PGDP) has a significant positive spatial spillover effect, which means that economic growth not only promotes the local carbon emission efficiency but also significantly enhances the carbon emission efficiency of neighboring areas. Urbanization (Urban) has a significant negative spatial spillover effect, indicating that urbanization development inhibits the improvement of carbon emission efficiency in peripheral regions. It may be due to the prosperity of core cities also bringing new opportunities for surrounding areas, and the influx of large population and industrial development increases energy consumption and carbon emission, which inhibits carbon emission efficiency. Under the geographic distance matrix, both population density (PopuD) and openness to the outside world (Open) have significant positive spatial spillover effects, indicating that the increase in population density or the expansion of the openness in provinces and cities not only enhances the local carbon emission efficiency but also promotes the neighboring one.

#### 4.2.4. Regression Results of Different Digital Economy Development Dimensions

In order to in-depth study the impact of different digital economy development dimensions on carbon emission efficiency in Chinese provinces and cities, this paper studies the regression results of four digital economy development indicators dimensions (as shown in Table 10). The spatial coefficients ρ all pass the significance level test of 5%, and there are significant differences in the impact and spatial spillover effects of different dimensions. Under the two matrices, the development of digital infrastructure (Infras), Internet development (IntDev), and digital finance (DF) have significant positive effects on local carbon emission efficiency, with the degree of influence ranging from low too high for DF, Infras, and IntDev. It may be due to the fact that digital infrastructure and Internet development, as the foundation of the hardware facilities and soft environment for the construction of digital economy, are an essential and continuously constructed part of the development of the digital economy. Therefore, they have a more powerful contribution to carbon emission efficiency as relatively mature digital economy development dimensions compared to digital finance, which is currently in the accelerated development stage.

The spatial spillover effects of different digital economy indicator dimensions are shown in Table 11. The results show that digital infrastructure has a positive spatial spillover effect, indicating that a well-developed digital infrastructure can increase the carbon emission efficiency of neighboring places. In addition, the digital industry and Internet development have positive spatial spillover effects under the adjacency matrix and geographic distance matrix, respectively, which means the in-depth expansion of the digital industry or Internet can help increase the carbon emission efficiency of neighboring regions.

### 4.3. Robustness Test

First, this paper performs robustness tests of the above results by replacing the different spatial weight matrixes and using the economic-geographic distance nested matrix and the inverse distance squared matrix to re-run the spatial econometric analysis. The empirical results in Table 12 show that the spatial coefficients under both matrices pass the significance tests, and the direction and significance of the estimated coefficients of Digital do not change significantly. In addition, the results for the control variables remain consistent with the previous paper. Therefore, the regression results remain stable under different spatial weight matrices.

Secondly, this paper refers to the endogeneity test method of You (2020) [64] and uses the first-order lagged term of the explanatory variable digital economic development (LDigital) to test the endogeneity problem between digital economic development and carbon emission efficiency. The results show that the spatial spillover effect of carbon emission efficiency remains significant, and the direction and significance of the coefficients of the explanatory and control variables do not change significantly. So, the conclusions of this paper are robust.

### 4.4. Further Research

China is a vast country with significant differences in digital economy development and energy resource endowment in diverse regions. In order to deeply analyze the regional heterogeneity of the impact of the digital economy on carbon emission efficiency, this paper divides the full sample of 30 Chinese provinces and cities into three sub-samples: the eastern region, the central region, and the western region, and conducts spatial econometric analysis separately.

The decomposition results of the spatial effect of the digital economy development (Digital) are shown in Table 13. The results show that the estimated coefficients of the direct effect of the digital economy are significantly positive in the eastern and central regions, indicating that the digital economy development has a significant contribution to the local carbon emission efficiency in the eastern and central, but not significant in the western. Meanwhile, the estimated coefficients show that the direct effect of the digital economy on carbon emission efficiency has significant regional heterogeneity, and the degree of impact of the digital economy on local carbon emission efficiency decreases from eastern to western regions. It may attribute to the fact that the development of the digital economy in western is relatively backward, and energy-consuming industries continue to flow in, which makes it difficult for the enhancement effect of the digital economy on carbon emission efficiency to play effectively in western regions. In addition, the reason why the results of the spatial spillover effect of the digital economy under the two spatial weight matrices differ significantly may be the spatial distribution characteristics of provinces and cities in different regions. For example, in eastern China, where the density of provinces and cities distribution is more concentrated than that in the central and western, the distribution of provincial and municipal borders and distances within the region is shown to be different from that of the full sample.

## 5. Discussion

In this paper, the findings of the above study are discussed as follows.

First, the digital economy development and carbon emission efficiency of Chinese provinces and cities are both spatially heterogeneous. According to the digital economy development index and carbon emission efficiency measured in this paper, the digital economy development of Chinese provinces and cities has an obvious distribution characteristic—strong in the east, weak in the middle and west. Sichuan province, the new highland of western development, has particularly outstanding development achievements of digital economy among western regions, basically in consonance with the study of Yu (2022) [15]. On the other hand, the carbon emission efficiency of Chinese provinces and cities shows evident unevenness and spatial correlation. Eastern China, where there are better underlying conditions and industrial structure, has a much higher carbon emission efficiency than the central and western regions. In addition, the carbon emission efficiency of Jiangxi, Hunan, Hubei, Sichuan, and Chongqing, located in the Yangtze River Economic Belt, is slightly higher than that of the rest of the central and western regions, which is basically in accord with the study of Jiang (2022) [65].

Second, the development of digital economy in Chinese provinces and cities has a facilitating effect on carbon emission efficiency, and there is a significant positive spatial spillover effect. The study by Xie (2022) [66] confirms that the level of digital economy development in Chinese provinces and cities is positively correlated with carbon emission efficiency in the industrial sector, which is consistent with the findings of this paper. The significant contribution digital economy development has on carbon emission efficiency may result from the following reasons. On the one hand, the digital economy development brings about changes in digital labor application and production and consumption patterns, which improve labor productivity and energy utilization and reduce labor and energy inputs as well as undesired carbon emission outputs. On the other hand, the digital economy, as a new economic development model, promotes high-quality economic development and increases economic efficiency output. Nonetheless, the indirect impact mechanism digital economy has on carbon emission efficiency remains to be further analyzed in subsequent studies.

Third, there are differences in the direct impact and spatial spillover effect of the development of the digital economy in different dimensions on the carbon emission efficiency of Chinese provinces and cities. Specifically, the development of digital finance, digital infrastructure, and the Internet has a significant positive impact on carbon emission efficiency from low to high. In addition, improved digital infrastructure construction, digital industry development, and Internet development help improve carbon emission efficiency in neighboring areas. This is in line with part of the findings of Xu (2022) [67], who confirmed that digital infrastructure reduces carbon emissions in adjacent areas. Meanwhile, Xu’s study shows that digital industry development has a significant negative effect on carbon emissions in both local and neighboring regions. However, this paper concludes that the development of the digital economy has not only a negative but insignificant effect on local carbon emission efficiency but also a significant positive effect on carbon emission efficiency in neighboring regions. The reasons for the above differences may be listed as follows. Firstly, the carbon emission efficiency index in this paper takes input and output factors into account rather than considering only the single impact of the digital economy on carbon emissions. Secondly, the measurement of the digital economy is not yet unified, and the different measuring methods of the development of the digital industry in the two articles lead to differences in the results.

Fourth, the direct effects of the digital economy development on carbon emission efficiency in Chinese provinces and cities have significant regional heterogeneity. The positive effect of the digital economy on local carbon emission efficiency decreases from the eastern to the western regions, and the effect in the western is not significant. A study by Xie (2022) [66] also pointed out that the digital economy contributes to carbon emission efficiency in eastern and central China, and the impact is more significant in eastern. However, Xie’s conclusion that “the digital economy has a significant inhibitory effect on carbon emission efficiency in western China” differs from this paper. Probably it is because Xie focused on the carbon emission efficiency of China’s industrial sectors. In addition, since the spatial distribution characteristics of provinces and cities in the three major regions of China differ from each other, the spatial spillover effect of the digital economy on carbon emission efficiency in the three has not yet been found in this paper. Future studies can explore this topic based on Chinese urban data.

## 6. Conclusions

Based on existing studies on the digital economy and carbon emission, this paper measures the digital economy development index and carbon emission efficiency of 30 Chinese provinces and cities from 2011–2019, using the entropy weight method and the Super-SBM-Undesirable Model, respectively. Moreover, with full consideration of geographical factors, the SDM model based on the adjacency matrix and the SAR model based on the geographical distance matrix is constructed. Meanwhile, this paper empirically analyzes the direct and indirect effects of digital economy development on the carbon emission efficiency of Chinese provinces and cities. In addition, the regional heterogeneity of the effects of digital economy development on carbon emission efficiency in eastern, central, and western regions of China is further analyzed in this paper. The main findings are as follows.

First, the digital economy development and carbon emission efficiency in Chinese provinces and cities are spatially heterogeneous, and both show the spatial distribution characteristics of stronger in the East and weaker in the Middle and West.

Second, the digital economy development in Chinese provinces and cities has a significant positive direct effect and a spatial spillover effect on carbon emission efficiency, i.e., the digital economy development facilitates both local and neighboring places’ carbon emission efficiency.

Third, there are differences in the direct and spatial spillover effects of various dimensions of the digital economy development on the carbon emission efficiency in Chinese provinces and cities. Specifically, Internet development, digital infrastructure, and digital financial development have significant positive effects on local carbon emission efficiency, with the effect degree ranging from high to low.

Fourth, the direct effect of the digital economy development on carbon emission efficiency in Chinese provinces and cities has significant regional heterogeneity. The facilitating effect of the digital economy on local carbon emission efficiency decreases from the eastern to the western but is not so significant in the western regions.

Based on the above findings, this paper recommends the following policy recommendations.

First, actively develop the digital economy to boost the achievement of carbon emission reduction targets. On the one hand, the government needs to accelerate the development of digital finance while continuously improving digital infrastructure and striving to achieve the comprehensive development of the digital economy in multiple dimensions. On the other hand, it should strengthen publicity and education, emphasize and establish the concept of green development, promote the in-depth integration of digital technology and production and lifestyle, and accelerate the formation of green production and green consumption patterns. If necessary, the government can utilize policy instruments to give full play to the role of the digital economy in promoting carbon emission reduction and its efficiency improvement.

Second, attention should be paid to the spatial spillover effect of the digital economy to enhance carbon emission efficiency. The development of the digital economy has realized the cross-domain connection of information, technology, talents, and other resources. Thus, sharing and cooperation has become an inevitable choice for regional development. Governments at all levels need to break down the barriers to information exchange and institutional mechanisms between provinces and cities. By establishing a coordination mechanism for inter-provincial linkage of digital economy development, the flow and allocation of digital technology and innovation resources between provinces and cities will be strengthened, and the radiation-driven function of strong provinces in digital economy development will be fully played.

Third, concerning the imbalance of digital economy development between provinces and cities, supporting and directing the construction and development of lagging areas. China‘s digital economy development resources and construction elements should be appropriately tilted to the central and western regions to create a favorable digital economy development environment for the lagging regions. Government departments should strive for the early realization of the late-mover advantage of the digital economy in the lagging development areas to boost carbon emission reduction by promoting the transfer of digital technology from developed eastern regions to the backward central and western regions, especially to the west with relatively lagging economic development and heavy industrial structure.

This paper provides not only new empirical evidence for the study of digital economy and carbon emission efficiency but also provides theoretical support and policy suggestions for developing countries such as China to actively develop digital economy to promote energy conservation and emission reduction and achieve green and low-carbon transformation. However, there are still shortcomings in this paper. First, this paper studies only the direct impact of the digital economy on carbon emission efficiency and its spatial spillover effect, with the indirect impact mechanism between the two uninvolved. Future research can further study the mediating role of energy intensity, industrial structure, and other factors. Secondly, there is no current standard in the measuring method and selection of indicators for the digital economy, and this paper measures only the digital economy indices of Chinese provinces and cities from four dimensions. Research can define and unify the digital economy and its measurement in the future.

## Figures and Tables

**Table 1:** Variables selection for the Super-SBM-Undesirable Model.

	Variables	Variable Definitions	Unit
Input	Labor Input	The number of employees	Million
	Capital Input	Capital stock calculated using 2011 as the base period	Billion Yuan
	Energy Input	The total energy consumption	Million Tons of Standard Coal
Desired Output	Economic Output	The real GDP calculated using 2011 as the base period	Billion Yuan
Undesired Output	Carbon Output	The total carbon dioxide emissions from all energy sources	Million Tons

**Table 2:** Digital Economy Development Index System.

Primary Indicators	Secondary Indicators	Tertiary Indicators	Weights	Indicator Direction
Digital Economy	Digital Infrastructure (Infras)	Length of fiber optic cable lines per capita	2.80%	+
		Number of Internet broadband access ports	3.78%	+
		Number of cell phone base stations	3.64%	+
		Number of Internet domain names	9.25%	+
	Internet Development (IntDev)	Mobile Phone Penetration Rate	1.70%	+
		Internet broadband access users	3.87%	+
	Digital Industry Development (IndusDev)	The output value of the information service industry	8.85%	+
		Total telecom business per capita	7.04%	+
		The total turnover of technology contracts	11.80%	+
		Software industry revenue	11.39%	+
		Percentage of employed persons in urban units of information transmission, software, and information technology service industry	5.56%	+
	Digital Finance (DF)	The breadth of digital financial coverage	25.27%	+
		Depth of digital finance usage	1.42%	+
		Digitalization of digital finance	1.78%	+
		Level of online mobile payment	1.85%	+

**Table 3:** Research variables and their definitions.

Variable Type	Variables	Variable Definitions
Explained variable	Carbon Emission Efficiency (CEE)	Carbon emission efficiency calculated based on the Super-SBM-Undesirable Model
Explanatory variable	Digital Economy (Digital)	The digital economy development index calculated based on the entropy method
Control variable	Population Density (PopuD)	The number of people per unit area
	Economic Development (PGDP)	dRegional GDP per capita
	Technological Innovation (RD)	Total internal expenditure on R&D
	Industry Structure (Indus)	The ratio of tertiary industry output to secondary industry
	Urbanization (Urban)	The ratio of the non-agricultural population to the total population

**Table 4:** Descriptive statistics of variables.

Variable Type	Variables	Obs	Mean	Std. Dev.	Min	Max
Explained variable	CEE	270	0.4505	0.2218	0.2281	1.2880
Explanatory variable	Digital	270	0.1156	0.0991	0.0104	0.5844
Control variable	PopuD	270	2864.2	1152.1	764.00	5821.0
	PGDP	270	54,717	26,320	16,413	164,220
	RD	270	872,444	1,667,339	196.00	1.20 × 10^7^
	Indus	270	1.1739	0.6664	0.5180	5.1692
	Open	270	178.53	264.83	0.1612	1522.9
	Urban	270	0.5764	0.1218	0.3496	0.8960

**Table 5:** Global Moran’s I of the carbon emission efficiency.

Year	W_binary_		W_distance_		W_distance2_		W_dis&eco_	
	Moran’s I	*p* Value	Moran’s I	*p* Value	Moran’s I	*p* Value	Moran’s I	*p* Value
2011	0.237	0.014 **	0.033	0.037 **	0.164	0.021 **	0.663	0.000 ***
2012	0.250	0.010 ***	0.039	0.025 **	0.168	0.019 **	0.660	0.000 ***
2013	0.214	0.024 **	0.028	0.052 *	0.138	0.044 **	0.647	0.000 ***
2014	0.232	0.016 **	0.035	0.033 **	0.151	0.030 **	0.638	0.000 ***
2015	0.215	0.024 **	0.030	0.047 **	0.138	0.044 **	0.630	0.000 ***
2016	0.212	0.025 **	0.031	0.045 **	0.139	0.042 **	0.629	0.000 ***
2017	0.218	0.021 **	0.034	0.034 **	0.148	0.031 **	0.634	0.000 ***
2018	0.230	0.017 **	0.038	0.025 **	0.160	0.024 **	0.631	0.000 ***
2019	0.241	0.016 **	0.045	0.018 **	0.179	0.015 **	0.624	0.000 ***

Note: *, **, *** indicate significant at 10%, 5% and 1% confidence level, respectively.

**Table 6:** The baseline regression results.

	(1)	(2)
Variables	OLS	Spatial Fixed Effect
Digital	0.212 ***	0.212 ***
	(4.52)	(4.52)
PopuD	1.81 × 10^−5^ ***	1.81 × 10^−5^ ***
	(4.56)	(4.56)
PGDP	1.96 × 10^−7^	1.96 × 10^−7^
	(0.74)	(0.74)
RD	−2.27 × 10^−9^	−2.27 × 10^−9^
	(−0.50)	(−0.50)
Indus	0.0139	0.0139
	(1.56)	(1.56)
Open	1.56 × 10^−4^ ***	1.56 × 10^−4^ ***
	(4.36)	(4.36)
Urban	0.148 *	0.148 *
	(1.84)	(1.84)
Constant	0.891 ***	0.236 ***
	(17.95)	(6.30)
Hausman Test		44.92 (0.0000)
Spatial fixed	Yes	Yes
R^2^	0.991	0.461
Observations	270	270

Note: T statistics in parentheses. *, *** indicate significant at 10%, and 1% confidence level, respectively.

**Table 7:** Spatial econometric model applicability tests.

	LM-Error	R LM-Error	LM-Lag	R LM-Lag	Wald-Lag	Wald-Error	LR-Lag	LR-Error
W_binary_	185.92 ***	161.67 ***	25.477 ***	1.245	5.56	23.79 ***	24.42 ***	30.36 ***
	(0.000)	(0.000)	(0.000)	(0.264)	(0.3512)	(0.0012)	(0.0004)	(0.0000)
W_distance_	396.03 ***	303.44 ***	100.73 ***	8.140 ***	7.00	15.77 **	19.90 ***	25.57 ***
	(0.000)	(0.000)	(0.000)	(0.004)	(0.2203)	(0.0273)	(0.0029)	(0.0003)
W_distance2_	255.13 ***	113.61 ***	155.87 ***	14.35 ***	5.21	24.51 ***	24.39 ***	31.10 ***
	(0.000)	(0.000)	(0.000)	(0.000)	(0.3911)	(0.0009)	(0.0004)	(0.0000)
W_dis&eco_	62.216 ***	34.301 ***	32.019 ***	4.104 **	45.47 ***	45.59 ***	44.44 ***	47.68 ***
	(0.000)	(0.000)	(0.000)	(0.043)	(0.0000)	(0.0000)	(0.0000)	(0.0000)

Note: *p*-value in parentheses. **, *** indicates significant at 5% and 1% confidence levels, respectively.

**Table 8:** Spatial econometric regression results.

Variables	SDM-W_binary_		SAR-W_distance_
	x	W * x	
Digital	0.161 ***	0.124 *	0.169 ***
	(3.64)	(1.90)	(3.74)
PopuD	1.12 × 10^−5^ ***	1.64 × 10^−7^	1.71 × 10^−5^ ***
	(3.02)	(0.02)	(4.76)
PGDP	4.61 × 10^−8^	1.54 × 10^−6^ ***	1.27 × 10^−7^
	(0.18)	(3.64)	(0.53)
RD	4.59 × 10^−10^	−9.95 × 10^−9^	−2.42 × 10^−9^
	(0.11)	(−1.46)	(−0.59)
Indus	0.0120	0.00758	0.00847
	(1.23)	(0.47)	(0.99)
Open	1.82 × 10^−4^ ***	2.43 × 10^−5^	1.29 × 10^−4^ ***
	(4.38)	(0.35)	(3.89)
Urban	0.144	−0.558 ***	0.209 **
	(0.91)	(−2.66)	(2.55)
ρ	0.195 **		0.454 ***
	(2.48)		(3.54)
sigma^2^	3.79 × 10^−4^ ***		4.20 × 10^−4^ ***
	(11.57)		(11.55)
LogL	679.0176		664.5960
R^2^	0.521		0.257
Observations	270		270

Note: Z statistics in parentheses. *, **, *** indicate significant at 10%, 5% and 1% confidence level, respectively.

**Table 9:** Decomposition results of spatial spillover effects.

Variables	SDM-W_binary_			SAR-W_distance_		
	Direct	Indirect	Total	Direct	Indirect	Total
Digital	0.169 ***	0.182 **	0.351 ***	0.166 ***	0.131 **	0.297 ***
	(3.76)	(2.58)	(4.09)	(3.68)	(2.15)	(3.41)
PopuD	1.12 × 10^−5^ ***	2.95 × 10^−6^	1.41 × 10^−5^	1.69 × 10^−5^ ***	1.36 × 10^−5^ **	3.05 × 10^−5^ ***
	(3.05)	(0.33)	(1.32)	(4.85)	(2.10)	(3.64)
PGDP	1.42 × 10^−7^	1.88 × 10^−6^ ***	2.02 × 10^−6^ ***	1.44 × 10^−7^	1.12 × 10^−7^	2.56 × 10^−7^
	(0.57)	(3.64)	(3.58)	(0.62)	(0.53)	(0.60)
RD	−6.72 × 10^−11^	−1.19 × 10^−8^	−1.20 × 10^−8^	−1.61 × 10^−9^	−1.28 × 10^−9^	−2.89 × 10^−9^
	(−0.02)	(−1.45)	(−1.16)	(−0.39)	(−0.33)	(−0.37)
Indus	0.0126	0.0128	0.0254	0.00397	0.00232	0.00630
	(1.35)	(0.67)	(1.24)	(0.49)	(0.34)	(0.44)
Open	1.87 × 10^−4^ ***	6.90 × 10^−5^	2.56 × 10^−4^ ***	1.51 × 10^−4^ ***	1.23 × 10^−4^ **	2.74 × 10^−4^ ***
	(4.60)	(0.92)	(3.25)	(4.61)	(2.01)	(3.43)
Urban	0.116	−0.644 ***	−0.528 ***	0.0598	0.0425	0.102
	(0.72)	(−2.84)	(−2.64)	(0.77)	(0.62)	(0.72)

Note: Z statistics in parentheses. **, *** indicate significant at 5% and 1% confidence level, respectively.

**Table 10:** Regression results for the four digital economy indicator dimensions.

Variables	SDM-W_binary_				SAR-W_distance_			
Infras	0.163 ***				0.140 ***			
	(5.34)				(4.71)			
IntDev		0.226 ***				0.242 ***		
		(5.56)				(6.66)		
IndusDev			−0.0355				0.0649	
			(−0.67)				(1.39)	
DF				0.0277 *				0.0276 *
				(1.70)				(1.69)
W*Infras	0.0618							
	(0.94)							
W*IntDev		0.0216						
		(0.34)						
W*IndusDev			0.219 ***					
			(3.05)					
W*DF				0.00974				
				(0.38)				
Controls	Yes	Yes	Yes	Yes	Yes	Yes	Yes	Yes
ρ	0.221 ***	0.189 **	0.192 **	0.274 ***	0.483 ***	0.384 ***	0.467 ***	0.536 ***
	(2.82)	(2.46)	(2.45)	(3.77)	(4.92)	(3.72)	(4.12)	(5.61)
Sigma^2^	3.62 × 10^−4^ ***	3.55 × 10^−4^ ***	3.89 × 10^−4^ ***	3.96 × 10^−4^ ***	3.99 × 10^−4^ ***	3.72 × 10^−4^ ***	4.29 × 10^−4^ ***	4.25 × 10^−4^ ***
	(11.56)	(11.58)	(11.57)	(11.53)	(11.57)	(11.59)	(11.56)	(11.55)
LogL	684.8198	688.0349	675.5984	671.8973	671.3763	681.4870	661.6433	662.0840
R^2^	0.540	0.552	0.511	0.482	0.500	0.540	0.453	0.438
Observations	270	270	270	270	270	270	270	270

Note: Z statistics in parentheses. *, **, *** indicate significant at 10%, 5% and 1% confidence level, respectively.

**Table 11:** Decomposition results of the spatial spillover effects of the four digital economy indicator dimensions.

Variables	SDM-W_binary_			SAR-W_distance_		
	Direct	Indirect	Total	Direct	Indirect	Total
Infras	0.169 ***	0.117 *	0.286 ***	0.144 ***	0.136 **	0.280 ***
	(5.42)	(1.67)	(3.62)	(4.66)	(2.24)	(3.60)
IntDev	0.230 ***	0.0706	0.301 ***	0.246 ***	0.156 **	0.401 ***
	(5.64)	(1.05)	(4.42)	(6.62)	(2.27)	(4.99)
IndusDev	−0.024	0.246 ***	0.222 ***	0.068	0.052	0.119
	(−0.46)	(3.18)	(3.07)	(1.39)	(1.19)	(1.39)
DF	0.029 *	0.022	0.052	0.030 *	0.034	0.063
	(1.71)	(0.68)	(1.24)	(1.68)	(1.31)	(1.54)

Note: Z statistics in parentheses. *, **, *** indicate significant at 10%, 5% and 1% confidence level, respectively.

**Table 12:** Robustness test results.

Variables	Replace the Spatial Weight Matrix			Explanatory Variables Lagged One Period		
	SDM-W_dis&eco_		SAR-W_distance_	SDM-W_dis&eco_		SAR-W_distance_
	x	W*x		W*x	x	
Digital	0.116 **	0.204 *	0.182 ***			
	(2.49)	(1.92)	(4.22)			
LDigital				0.127 **	0.0632	0.164 ***
				(2.29)	(0.67)	(3.09)
PopuD	1.30 × 10^−5^ ***	−1.56 × 10^−5^	1.70 × 10^−5^ ***	9.55 × 10^−6^ **	8.78 × 10^−6^	1.36 × 10^−5^ ***
	(3.67)	(−1.20)	(4.74)	(2.49)	(1.10)	(3.70)
PGDP	−5.77 × 10^−7^ **	1.24 × 10^−6^ **	1.04 × 10^−7^	−2.92 × 10^−7^	1.68 × 10^−6^ ***	−1.69 × 10^−7^
	(−2.01)	(2.17)	(0.43)	(−1.09)	(3.78)	(−0.68)
RD	8.20 × 10^−9^ *	6.17 × 10^−9^	−2.31 × 10^−9^	1.12 × 10^−10^	−9.12 × 10^−9^	−4.14 × 10^−10^
	(1.80)	(0.67)	(−0.56)	(0.02)	(−1.21)	(−0.09)
Indus	0.0196 *	0.0107	0.00531	0.0123	0.0222	0.00701
	(1.91)	(0.58)	(0.63)	(1.20)	(1.28)	(0.80)
Open	2.16 × 10^−4^ ***	7.77 × 10^−5^	1.38 × 10^−4^ ***	1.40 × 10^−4^ ***	3.54 × 10^−5^	1.13 × 10^−4^ ***
	(6.20)	(1.26)	(4.23)	(3.23)	(0.49)	(3.26)
Urban	0.788 ***	−1.171 ***	0.101	0.116	−0.325	0.141 *
	(4.74)	(−5.87)	(1.37)	(0.72)	(−1.47)	(1.71)
ρ	0.234 **		0.289 ***	0.203 **		0.497 ***
	(2.57)		(3.48)	(2.51)		(4.74)
sigma^2^	3.59 × 10^−4^ ***		4.17 × 10^−4^ ***	3.26 × 10^−4^ ***		3.52 × 10^−4^ ***
	(11.55)		(11.56)	(10.91)		(10.90)
LogL	686.1460		665.3780	621.5362		611.6252
R^2^	0.543		0.475	0.548		0.512
Observations	270		270	240		240

Note: Z statistics in parentheses. *, **, *** indicate significant at 10%, 5% and 1% confidence level, respectively.

**Table 13:** Results of regional heterogeneity analysis.

Digital	SDM-W_binary_			SAR-W_distance_		
	East	Middle	West	East	Middle	West
Direct	0.199 ***	0.108 ***	0.097	0.252 ***	0.161 ***	0.047
	(2.64)	(3.09)	(1.10)	(3.45)	(3.75)	(0.47)
Indirect	0.105	0.089 *	0.451 *	0.104	0.041	0.010
	(1.04)	(1.83)	(1.87)	(1.40)	(1.24)	(0.20)
Total	0.304 **	0.197 ***	0.547 *	0.356 ***	0.202 ***	0.057
	(2.21)	(3.00)	(1.84)	(2.91)	(3.81)	(0.40)

Note: Z statistics in parentheses. *, **, *** indicate significant at 10%, 5% and 1% confidence level, respectively.

## Data Availability

Not applicable.
